# Public Policy and Aging Report

**DOI:** 10.1093/geroni/igaf122.1237

**Published:** 2025-12-31

**Authors:** Michael Lepore

**Affiliations:** University of Massachusetts Amherst, Amherst, Massachusetts, United States

## Abstract

All manuscripts submitted to Public Policy & Aging Report should address practice and/or public policy implications and aging. The journal publishes three types of articles. (1) Policy Analysis Articles. Public Policy & Aging Report is committed to publishing high quality analyses of public policy and aging issues. Original research is welcome, or articles can provide expert analysis of and commentary on key policy issues drawing on past studies. (2) Policy Spotlight Articles. Public Policy & Aging Report is committed to raising awareness of important public policy and aging issues. The goal of Policy Spotlight Articles is to provide a short overview of key policy issues. (3) Point-Counterpoint Articles. Public Policy & Aging Report is committed to heightening scholarly engagement with key public policy and aging issues. The goal of Point-Counterpoint Articles is to engage different scholarly perspectives on public policy and aging issues addressed in Public Policy & Aging Report. Point-Counterpoint Articles should respond to and reference one or more article(s) in a prior issue of Public Policy & Aging Report and should provide alternate perspectives on the topic including a clear statement of the counterargument.

